# Widening the phenotypic spectrum caused by pathogenic PDX1 variants in individuals with neonatal diabetes

**DOI:** 10.1136/bmjdrc-2024-004439

**Published:** 2024-11-14

**Authors:** Nicola Jeffery, Omar Al Nimri, Jayne A L Houghton, Evgenia Globa, Matthew N Wakeling, Sarah E Flanagan, Andrew T Hattersley, Kashyap Amratlal Patel, Elisa De Franco, On behalf of PDX1-NDM consortium, P K Varthakavi

**Affiliations:** 1 Department of Clinical and Biomedical Sciences, University of Exeter Faculty of Health and Life Sciences, Exeter, UK; 2 Exeter Genomics Laboratory, Royal Devon University Healthcare NHS Foundation Trust, Exeter, UK; 3 Ukrainian Scientific and Practical Center of Endocrine Surgery, Transplantation of Endocrine Organs and Tissues of the Ministry of Health of Ukraine, Kyiv, Ukraine; 4 Diabetes and Endocrinology, Royal Devon and Exeter NHS Foundation Trust, Exeter, UK

**Keywords:** Genetics

## Abstract

**Introduction:**

Biallelic *PDX1* variants are a rare cause of isolated pancreatic agenesis and neonatal diabetes (NDM) without exocrine pancreatic insufficiency, with 17 cases reported in the literature.

**Research design and methods:**

To determine the phenotypic variability caused by this rare genetic aetiology, we investigated 19 individuals with NDM resulting from biallelic disease-causing *PDX1* variants.

**Results:**

Of the 19 individuals, 8 (42%) were confirmed to have exocrine insufficiency requiring replacement therapy. Twelve individuals (63.2%) had extrapancreatic features, including 8 (42%) with conditions affecting the duodenum and/or hepatobiliary tract. Defects in duodenum development are consistent with previous *Pdx1* ablation studies in mice which showed abnormal rostral duodenum development.

**Conclusions:**

Our findings show that recessive *PDX1* variants can cause a syndromic form of NDM, highlighting the need for clinical assessment of extrapancreatic features in individuals with NDM caused by *PDX1* variants.

**WHAT IS ALREADY KNOWN ON THIS TOPIC**

- Biallelic *PDX1* variants are a rare genetic cause of diabetes diagnosed in the first 6 months of life (neonatal diabetes, NDM).
- Initially, recessive loss-of-function *PDX1* variants were identified as a cause of pancreatic agenesis; later studies expanded the pancreatic phenotype to include NDM with or without subclinical exocrine insufficiency.
- Recent reports have described extrapancreatic features in three unrelated cases with *PDX1*-NDM variants, highlighting the need to comprehensively assess the clinical phenotype caused by these variants.

**WHAT THIS STUDY ADDS**

- In our cohort of 19 individuals with *PDX1*-NDM, extrapancreatic features were common (detected in 63.2% of patients), with defects affecting the duodenum and/or hepatobiliary tract being present in 8 individuals (42% of the cohort).

**HOW THIS STUDY MIGHT AFFECT RESEARCH, PRACTICE OR POLICY**

- Our findings highlight the need for clinical assessment of extrapancreatic features in individuals with *PDX1*-NDM.

## Introduction

 Diabetes diagnosed before the age of 6 months (neonatal diabetes, NDM) is a genetically and clinically heterogeneous condition, with disease-causing variants in 1 of >35 known causative genes identified in >85% of cases.^1^ Pathogenic variants in >25 genes cause syndromic forms of NDM, each associated with specific extrapancreatic features affecting a range of organs.^2^ Clinical management of these individuals is often challenging due to the rarity of the conditions, heterogeneity of clinical features, disease severity and lack of specific treatments. The identification and assessment of individuals with syndromic NDM is essential to define the phenotype, guide clinical management, and inform on prognosis and recurrence risk within families.

Biallelic *PDX1* variants are a rare genetic cause of NDM, with 17 cases reported worldwide.^3^,^12^ Initially, recessive loss-of-function *PDX1* variants were identified as a cause of pancreatic agenesis without extrapancreatic features in three unrelated families.^8^,^10^ Later studies expanded the pancreatic phenotype to include NDM with or without subclinical exocrine insufficiency.^3 6^ Recent reports have described extrapancreatic features in three unrelated cases with homozygous *PDX1* variants,^4 5 7^ highlighting the need to comprehensively assess the clinical phenotype caused by these variants.

In this study we assessed the genetic and clinical features of 19 individuals with biallelic *PDX1* variants to define the pancreatic and extrapancreatic features of this NDM subtype.

## Research design and methods

### Subjects and genetic analysis

The study complied with the Declaration of Helsinki. Patients’ parents or guardians gave their informed written consent.

Individuals with biallelic *PDX1* variants classified as pathogenic or likely pathogenic according to the American College of Medical Genetics and Genomics (ACMG) guidelines^13^ (n=19 from 17 families) were selected from an international cohort of 2601 individuals referred for NDM testing to the Exeter Genomics Laboratory. Genetic analysis of the *PDX1* gene (NM_000209.3) was performed by Sanger sequencing or targeted next-generation sequencing as previously described.^14^ Variant testing for family members was performed by Sanger sequencing. No functional assessment of the variants was performed as part of this study.

Consanguinity had been reported in 13/17 families. Eight patients had been included in previous publications, including the three reports describing individuals with extrapancreatic features.^3^,^57 15^

Haplotype analysis was performed using off-target reads from next-generation sequencing data using a software developed inhouse (https://github.com/rdemolgen/SavvySuite) in individuals I-4, I-5, and I-6 (who were homozygous for the p.(Ala152Gly) variant).

### Assessment of clinical phenotype

Clinical details at diagnosis were collected through a standardised NDM referral form with follow-up information collected for 14 individuals following email contact with the clinicians. The average age at last assessment was 5.3 years (range 2 months to 16 years). Given the difficulties in obtaining abdominal imaging in this international cohort, we clinically defined pancreatic agenesis/hypoplasia as insulin-treated NDM and exocrine insufficiency requiring replacement therapy.^16^

The χ^2^ test was used to assess associations between extrapancreatic features and variant type (missense vs stop gain/start loss/frameshift variants). Three individuals from family 2 who were compound heterozygotes for a missense and a frameshift protein-truncating variant were excluded from this analysis since they harboured both types of variants.

## Results

**Figure 1:**
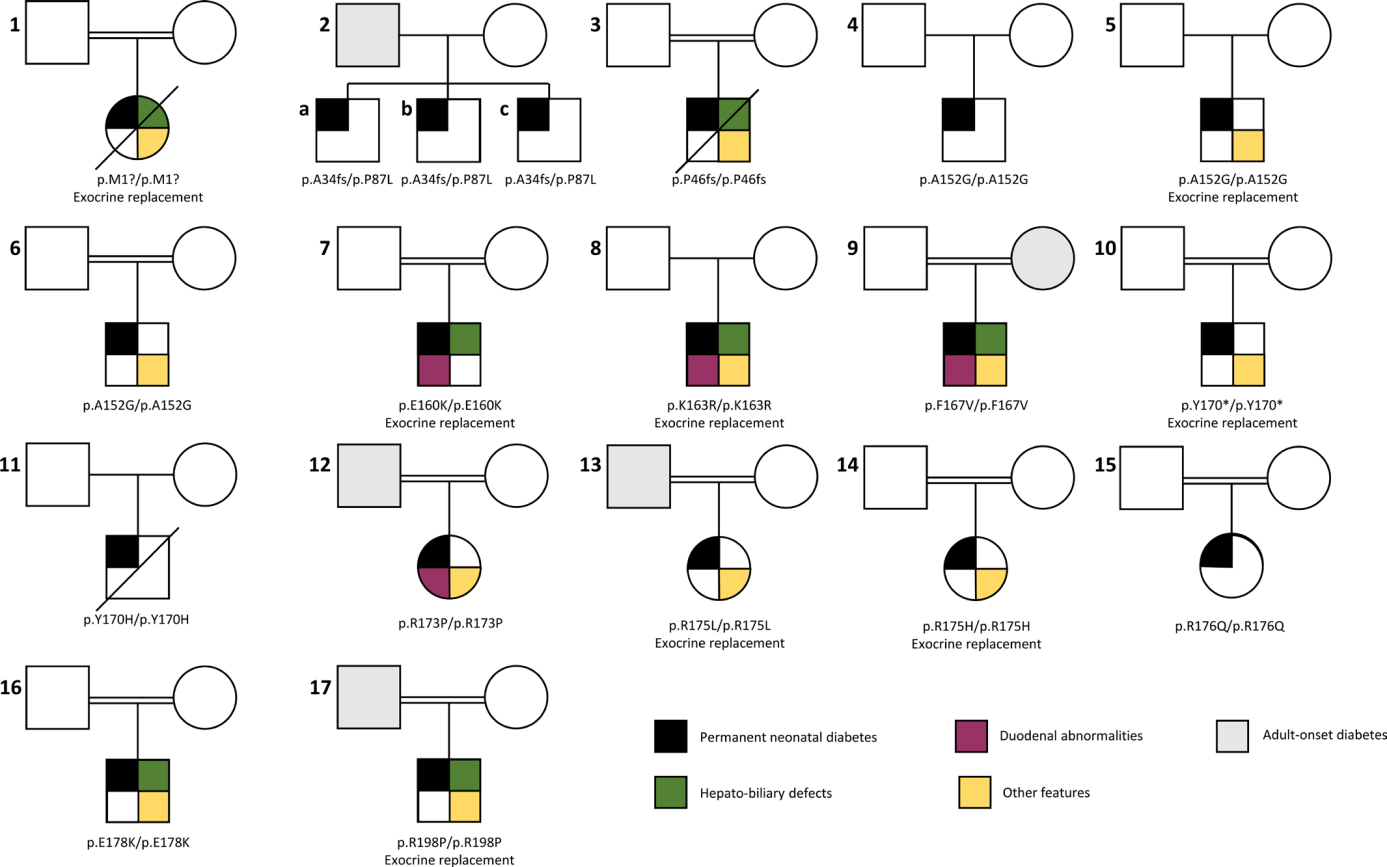
Partial pedigrees of the 17 families reported in this study with biallelic *PDX1* pathogenic variants. The variant is shown underneath each pedigree, along with information on whether the patient was on exocrine replacement therapy, consistent with a clinical diagnosis of pancreatic agenesis/hypoplasia. Key shown at the bottom right of the figure.

We identified 16 different *PDX1* variants, including 2 frameshifts, 1 start-loss, 1 stop-gain, and 12 missense, in 19 individuals with NDM (16 singletons and 3 affected siblings) (figures1  2, online supplemental tables 1 and 2).

**Figure 2:**
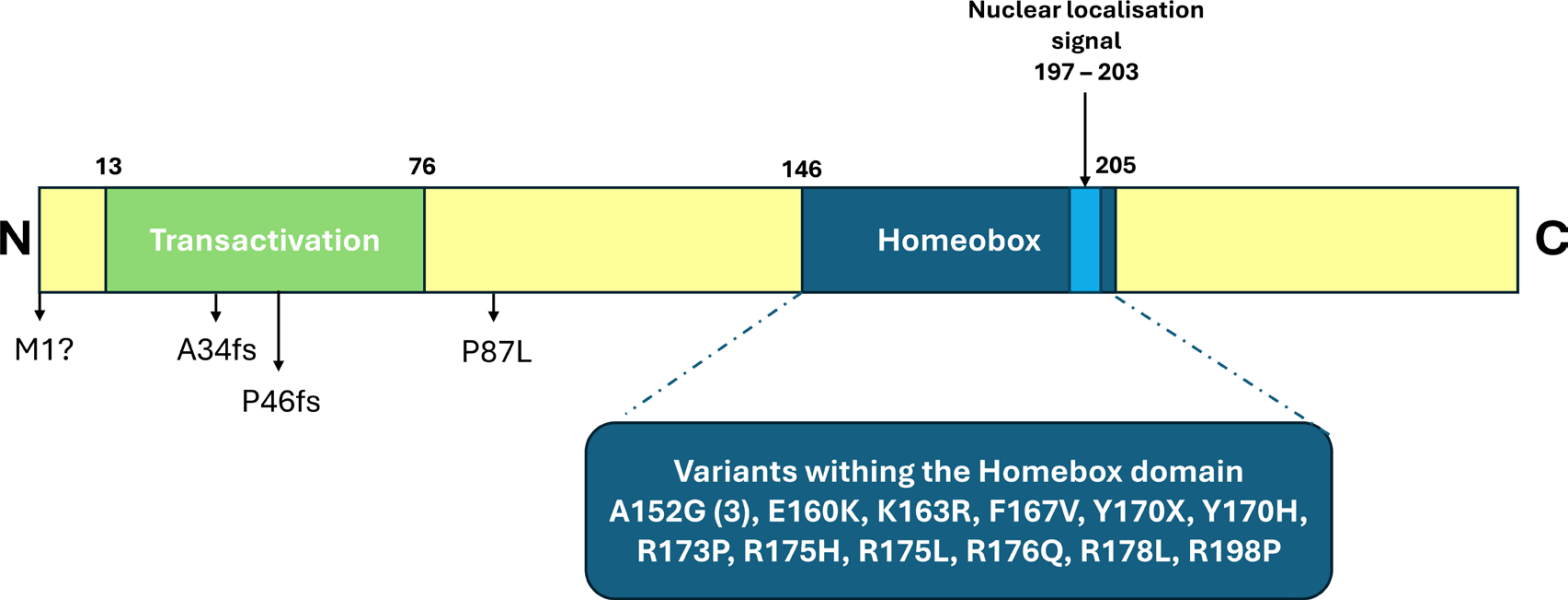
Variant location within the PDX1 protein. This schematic shows the location of each variant mapped to the PDX1 protein. The location of the protein domains is shown as reported by UniProt (https://www.uniprot.org/). The transactivation domain is essential for transactivation of promoters by PDX1, including the *INS* gene promoter in beta cells.^24^ The highly conserved homeobox domain mediates DNA binding and contains the nuclear localisation signal. Only one of the missense variants identified in our study affects a residue within the Homeobox domain which is also part of the nuclear localisation signal (p.R198P).

All but one of the missense variants, p.(Pro87Leu), affected a residue within the homeobox domain of the protein. The variants were homozygous in all cases except for family 2, where the three affected siblings were compound heterozygotes for a missense and a frameshift variant (p.(Ala34fs)/p.(Pro87Leu)).

Patients I-4, I-5, and I-6 were homozygous for the same missense variant, p.(Ala152Gly), despite no known family relationship. Haplotype analysis detected a shared ~4 Mb haplotype on chromosome 13 including *PDX1,* consistent with a common distant ancestor.

### Pancreatic phenotype

All 17 individuals for whom birth weight data were available were born small for gestational age (median birth weight *z*-score −3.09; range: −4.51 to −1.65). The diabetes was permanent in all cases, with a median age at diagnosis of 20 days (range: 1–181 days, data available for 19/19 individuals). All patients were insulin-treated (median dose 0.93, range 0.2–2.5 U/kg/day at last assessment, data available for 16 individuals).

In eight individuals, NDM and exocrine pancreatic insufficiency requiring replacement therapy was consistent with a clinical diagnosis of pancreatic agenesis/hypoplasia. Fecal elastase measurement was normal (>200 µg) for two of the three affected siblings from family 2 (I-2a and I-2b), suggesting normal exocrine function. Exocrine pancreatic insufficiency was not biochemically investigated in the remaining nine individuals (including the third affected sibling of family 2, I-2c). Clinical symptoms of exocrine insufficiency were reported in only one of these individuals, I-9, who had vitamin D malabsorption and a BMI less than the first centile. Abdominal imaging in this individual showed an annular pancreas.

### Extrapancreatic features

Twelve patients had at least one extrapancreatic feature (63.2% of the cohort at last assessment), (online supplemental table 1). Developmental abnormalities affecting the duodenum, gallbladder, and hepato-biliary system were most common (8/19, 42%) either in combination or individually. Rarer extrapancreatic features included anemia (n=4), developmental delay (n=4), dysmorphic features (n=3), heart defects (n=2), structural kidney defects (n=2), and epilepsy (n=2).

Three individuals died before 1 year of age. I-1 and I-3, who were homozygous for variants predicted to result in complete loss of PDX1, died of sepsis following multiorgan failure and worsening liver function, respectively. The cause of death was not known in I-11 who was homozygous for a missense variant affecting a residue within the homeobox domain.

### Genotype-phenotype relationship

No association was observed between pancreatic exocrine insufficiency, duodenal/hepatobiliary features, or all extrapancreatic features, and variant type (frameshift/stop-gain/stop-loss vs missense, p=1 for all three conditions).

## Discussion

Our study of 19 individuals with *PDX1*-NDM broadens the phenotype associated with this condition, with 42% of cases having extrapancreatic features affecting the duodenum, gallbladder, and/or hepato-biliary system.

Our results confirm that variability in the pancreatic phenotype exists between individuals with biallelic *PDX1* variants. These variants were originally reported to cause pancreatic agenesis, with subsequent studies identifying patients diagnosed with NDM with either subclinical or no exocrine pancreatic insufficiency.^36 8^,^10^ In our cohort, 8/19 patients had NDM and biochemically confirmed exocrine pancreatic insufficiency consistent with pancreatic agenesis/hypoplasia, while 2 individuals (siblings) had no biochemical or clinical evidence of exocrine pancreatic insufficiency (previously reported^3^). For the nine remaining patients, exocrine pancreatic function was not assessed. While exocrine insufficiency was not clinically suspected in eight of these individuals, in the absence of biochemical tests we cannot exclude the possibility that some had subclinical exocrine insufficiency. Confirming pancreatic exocrine insufficiency in these patients is important because, if untreated, it can lead to impaired growth.

Developmental abnormalities of the duodenum, gallbladder, and hepatobiliary system were identified in 8/19 (42%) individuals in our cohort. These features are consistent with mouse models where *Pdx1* loss results in abnormal development of the pancreas and rostral duodenum.^17^ In humans, a recent study showed strong reduction of enteroendocrine cells in intestinal organoids differentiated from induced pluripotent stem cells collected from a patient with pancreatic agenesis and chronic diarrhoea caused by a homozygous p.(Pro63fs) *PDX1* variant.^18^ The observation of duodenum and hepatobiliary developmental abnormalities in almost half of our patients, together with the evidence provided by in vivo and in vitro studies, confirm the critical role of *PDX1* in development of the pancreas, duodenum, and hepatobiliary tract.

The phenotypic spectrum observed in our patients with *PDX1*-NDM overlaps with features seen in Mitchell-Riley syndrome caused by recessive *RFX6* variants (OMIM:615710). In vitro studies in human embryonic stem cells have shown that PDX1 and RFX6 regulate each other’s expression during pancreatic development. RFX6 binds to an enhancer 10 kb upstream of the *PDX1* transcription start site at the pancreatic gut tube differentiation stage^19^ and PDX1 binds to a putative pancreatic progenitor enhancer ~1 kb downstream of *RFX6*.^20^ The overlapping phenotype observed in *PDX1*-NDM and *RFX6*-NDM suggests that these two transcription factors share important regulatory roles in the development of other endodermal organs, in addition to the pancreas.

Genotype-phenotype relationships have been suggested for some *PDX1* variants. Heterozygous variants are associated with type 2 diabetes,^21^ the dominant negative p.(Pro63fs) variant was identified in a large pedigree with Maturity-onset diabetes of the young (MODY),^22 23^ and recessive variants cause pancreatic agenesis^8^,^10^ or NDM.^3 6^ The p.(Pro87Leu) variant identified in family 2 in this study has been suggested to be a hypomorphic variant.^3^ Consistent with this, individuals I-2a and I-2b were the only individuals in our cohort with normal fecal elastase measurement and none of the three siblings in family 2 have extrapancreatic features. Furthermore, I-4, I-5, and I-6, who shared the same homozygous variant, did not have hepatobiliary or duodenal defects which were otherwise common in the cohort, suggesting the possibility that the genotype affects the manifestation of these extrapancreatic features. Despite this, we did not find a significant association between the type of variant (missense vs stop-gain/start-loss/frameshift) and the presence of extrapancreatic features or exocrine insufficiency when assessing the variants as a group. However, our power to detect an association was limited by the size of our cohort.

In conclusion, our findings show that *PDX1* recessive variants can result in syndromic NDM, providing further insights into the role of this transcription factor in human development. Understanding the broader phenotypic spectrum caused by *PDX1* recessive variants is essential for managing patients with this condition.

## Supplementary material

10.1136/bmjdrc-2024-004439 online supplemental table 1

## Data Availability

All data relevant to the study are included in the article or uploaded as supplementary information.
