# Supplementary material for: Widening the phenotypic spectrum caused by pathogenic PDX1 variants in individuals with neonatal diabetes
Source: BMJ Open Diabetes Res Care. 2024 Nov 14;12(6):e004439. doi: 10.1136/bmjdrc-2024-004439 (PMC11575358; doi:10.1136/bmjdrc-2024-004439)
Supplement: online supplemental table 1 [file bmjdrc-12-6-s001.pdf]

Supplementary Table 1: Genetic and clinical features of the 19 individuals with PDX1-neonatal diabetes. Abbreviations: F (Female), M (Male), EBV (Epstein–Barr virus), CMV (Cytomegalovirus), ASD (Atrial Septal Defect), BMI (Body Mass Index). References are provided for previously reported patients; all other patients have not been previous reported. Age at last assessment (which is different from current age in all cases) is indicated. All variants were heterozygous except for those in I-2a, I-2b and I-2c who were compound heterozygous.

ID	Age at last assessment	Reported consanguinity	Recessive *PDX1* variant	Birth weight/ gestation (z score)	Age at diabetes diagnosis (days)	Biochemically-confirmed exocrine pancreatic insufficiency	BMI centile at last assessment	Duodenal defect	Hepato-biliary defect	Other features	Parents diagnosed with diabetes	Reference
1	6 months (deceased)	Yes	c.1A>G, p.(Met1?)	1250 g/ 35 weeks (-2.94)	6	Confirmed	N/A	None reported	Intrahepatic bile ducts and gall bladder hypoplasia, elevated bilirubin levels, cytolysis syndrome, hepatomegaly, hepatolienal syndrome, cholestatic hepatitis, jaundice	Cardiac defect (accessory chord left ventricle), anaemia, suspected uretro-hydronephrosis. Positive for EBV and CMV. Cause of death was sepsis and multiorgn failure.	No	(1)

ID	Age at last assessment	Reported consanguinity	Recessive *PDX1* variant	Birth weight/ gestation (z score)	Age at diabetes diagnosis (days)	Biochemically-confirmed exocrine pancreatic insufficiency	BMI centile at last assessment	Duodenal defect	Hepato-biliary defect	Other features	Parents diagnosed with diabetes	Reference
2a	16 years	No	c.98dup/c.260C>T, p.(Ala34Cysfs*191)/ p.(Pro87Leu)	2500 g/ 40 weeks (-2.46)	18	Excluded (Fecal elastase normal)	57th	None	None	None	Yes (Father, Age at diagnosis: N/A, Treatment: Insulin)	(2)
2b	15 years	No	c.98dup/c.260C>T, p.(Ala34Cysfs*191)/ p.(Pro87Leu)	2760 g/ 40 weeks (-1.82)	18	Excluded (Fecal elastase normal)	66th	None	None	None	Yes (Father, Age at diagnosis: N/A, Treatment: Insulin)	(2)
2c	10 years	No	c.98dup/c.260C>T, p.(Ala34Cysfs*191)/ p.(Pro87Leu)	2400 g/ 38 weeks (-1.75)	7	Not tested	50th	None	None	None	Yes (Father, Age at diagnosis: N/A, Treatment: Insulin)	
3	2 months (deceased)	Yes	c.122_134 dup, p.(Pro46Alafs*183)	1360 g/ 34 weeks (-2.15)	6	Not tested	N/A	None	Biliary atresia, absent bile ducts, jaundice	Anaemia requiring transfusions, failure to thrive, exfoliative dermatitis. Cause of death was sepsis.	No	
4	9 months (referral)	Yes	c.455C>G, p.(Ala152Gly)	1750g/	2	Not tested	N/A	None	None	None	No	

ID	Age at last assessment	Reported consanguinity	Recessive *PDX1* variant	Birth weight/ gestation (z score)	Age at diabetes diagnosis (days)	Biochemically-confirmed exocrine pancreatic insufficiency	BMI centile at last assessment	Duodenal defect	Hepato-biliary defect	Other features	Parents diagnosed with diabetes	Reference
				40 weeks (-4.51)								
5	8 years	No	c.455C>G, p.(Ala152Gly)	1397g/ 37 weeks (-3.79)	114	Confirmed	1st	None	None	Bilateral cataracts	No	(2, 3)
6	3 years	Yes	c.455C>G, p.(Ala152Gly)	1750 g/ 35 weeks (-1.76)	28	Not tested	18th	None	None	Anaemia, septic arthritis	No	
7	7 years	Yes	c.478G>A, p.(Glu160Lys)	1800 g/ 38 weeks (-3.28)	181	Confirmed	N/A	Duodenal atresia	Gall bladder hypoplasia, elevated bilirubin	None	No	
8	8 years	No	c.488A>G, p.(Lys163Arg)	1260 g/ ? weeks	43	Confirmed	26th	Duodenal atresia	Gall bladder hypoplasia	Anaemia, nephrocalcinosis, nephropathy, developmental delay, dysmorphic features	No	(4)
9	6 years	Yes	c.499T>G, p.(Phe167Val)	1745 g/ 38 weeks (-3.43)	20	Not tested	<1st	Duodenal atresia	Cholestasis, hepatic insufficiency	Annular pancreas	Yes (Mother diagnosed at 29, Treatment: Diet)	(5)
10	6 months (referral)	Yes	c.510C>A, p.(Tyr170*)	1660 g/	11	Confirmed	N/A	None	None	None	No	

ID	Age at last assessment	Reported consanguinity	Recessive *PDX1* variant	Birth weight/ gestation (z score)	Age at diabetes diagnosis (days)	Biochemically-confirmed exocrine pancreatic insufficiency	BMI centile at last assessment	Duodenal defect	Hepato-biliary defect	Other features	Parents diagnosed with diabetes	Reference
				37 weeks (-3.09)								
11	5 months (deceased)	No	c.508T>C, p.(Tyr170His)	1500g/ 39 weeks (-4.70)	50	Not tested	N/A	None	None	None	No	(6)
12	6 years (referral)	Yes	c.518G>C, p.(Arg173Pro)	2100 g/ 40 weeks (-3.19)	58	Not tested	36th	Duodenal web	None	Epilepsy	Yes (Father diagnosed at 39, Treatment: OHA)	
13	6 years	Yes	c.524G>T, p.(Arg175Leu)	1300 g/ 37 weeks (-4.23)	14	Confirmed	87th	None	None	Developmental delay, dysmorphic features, growth delay	Yes (Father, age at diagnosis N/A, Treatment: Insuliln + OHA)	
14	7 years	Yes	c.524G>A, p.(Arg175His)	1400 g/ 34 weeks (-1.86)	1	Confirmed	N/A	None	None	Cardiac defect (secundum ASD), dysmorphic features, immune deficiency	No	
15	3 years (referral)	Yes	c.527G>A, p.(Arg176Gln)	1700g/ ? weeks	20	Not tested	23rd	None	None	None	No	(2, 3)

ID	Age at last assessment	Reported consanguinity	Recessive *PDX1* variant	Birth weight/ gestation (z score)	Age at diabetes diagnosis (days)	Biochemically-confirmed exocrine pancreatic insufficiency	BMI centile at last assessment	Duodenal defect	Hepato-biliary defect	Other features	Parents diagnosed with diabetes	Reference
16	5 years	Yes	c.532G>A, p.(Glu178Lys)	2440 g/ 38 weeks (-1.65)	73	Not tested	3rd	None	Dilated bile duct	Epilepsy, learning difficulties, developmental delay, dysmorphic features	No	
17	3 weeks (referral)	Yes	c.593G>C, p.(Arg198Pro)	1520 g/ 37 weeks (-3.46)	21	Confirmed	N/A	None	Gall bladder hypoplasia, cholestasis	Congenital hypothyroidism	Yes (Father, age at diagnosis 28 years, Treatment: N/A)	

Supplementary Table 2: Variant classification according to ACMG guidelines (7). PVS = Very strong, PS = Strong, PM = Moderate, PP = Supporting. When evidence was used with a different weight from its code, it is followed by _St (Strong), _M (Moderate) or _S (Supporting). PP4 was used only for patients with confirmed exocrine pancreatic insufficiency.

***PDX1* variant**	**Evidence**	**Classification**	**ACGS points (8)**
c.1A>G, p.(Met1?)	PVS1_M, PM2, PM3_S, PP4	LP	6
c.98dup, p.(Ala34Cysfs*191)	PVS1_St, PM2, PP1	LP	7
c.260C>T, p.(Pro87Leu)	PM2, PM3, PP1,PP3	LP	6
c.122_134 dup, p.(Pro46Alafs*183)	PVS1_St, PM2, PM3_S	LP	7
c.455C>G, p.(Ala152Gly)	PS4_M, PM1, PM2, PM3_S, PP3, PP4	LP	9
c.478G>A, p.(Glu160Lys)	PM1, PM2, PM3_S, PP3, PP4	LP	7
c.488A>G, p.(Lys163Arg)	PM1, PM2, PM3_S, PP3, PP4	LP	7
c.499T>G, p.(Phe167Val)	PM1, PM2, PM3_S, PP3	LP	6
c.510C>A, p.(Tyr170*)	PVS1_St, PM2, PM3_S, PP4	LP	8
c.508T>C, p.(Tyr170His)	PM1, PM2, PM3_S, PP3	LP	6
c.518G>C, p.(Arg173Pro)	PM1, PM2, PM3_S, PP3	LP	6
c.524G>T, p.(Arg175Leu)	PM1, PM2, PM3_S, PP3, PP4	LP	7
c.524G>A, p.(Arg175His)	PM1, PM2, PM3_S, PP3, PP4	LP	7
c.527G>A, p.(Arg176Gln)	PM1, PM2, PM3_S, PP3	LP	6
c.532G>A, p.(Glu178Lys)	PM1, PM2, PM3_S, PP3	LP	6
c.593G>C, p.(Arg198Pro)	PM1, PM2, PM3_S, PP3, PP4	LP	7

Supplementary References

1. Globa E, Zelinska N, Johnson MB, Flanagan SE, De Franco E. Neonatal and early-onset diabetes in Ukraine: Atypical features and mortality. Diabet Med. 2023;40(5):e15013.

2. De Franco E, Shaw-Smith C, Flanagan SE, Edghill EL, Wolf J, Otte V, et al. Biallelic PDX1 (insulin promoter factor 1) mutations causing neonatal diabetes without exocrine pancreatic insufficiency. Diabet Med. 2013;30(5):e197-200.

3. Flanagan SE, De Franco E, Lango Allen H, Zerah M, Abdul-Rasoul MM, Edge JA, et al. Analysis of transcription factors key for mouse pancreatic development establishes NKX2-2 and MNX1 mutations as causes of neonatal diabetes in man. Cell Metab. 2014;19(1):146-54.

4. Kulkarni A, Sharma VK, Nabi F. PDX1 Gene Mutation with Permanent Neonatal Diabetes Mellitus with Annular Pancreas, Duodenal Atresia, Hypoplastic Gall Bladder and Exocrine Pancreatic Insufficiency. Indian Pediatr. 2017;54(12):1052-3.

5. Sahebi L, Niknafs N, Dalili H, Amini E, Esmaeilnia T, Amoli M, et al. Iranian neonatal diabetes mellitus due to mutation in PDX1 gene: a case report. J Med Case Rep. 2019;13(1):258.

6. Ellard S, Lango Allen H, De Franco E, Flanagan SE, Hysenaj G, Colclough K, et al. Improved genetic testing for monogenic diabetes using targeted next-generation sequencing. Diabetologia. 2013;56(9):1958-63.

7. Richards S, Aziz N, Bale S, Bick D, Das S, Gastier-Foster J, et al. Standards and guidelines for the interpretation of sequence variants: a joint consensus recommendation of the American College of Medical Genetics and Genomics and the Association for Molecular Pathology. Genet Med. 2015;17(5):405-24.

8. https://www.acgs.uk.com/media/12533/uk-practice-guidelines-for-variant-classification-v12-2024.pdf
